# High-throughput sequencing identification of differentially expressed microRNAs in metastatic ovarian cancer with experimental validations

**DOI:** 10.1186/s12935-020-01601-4

**Published:** 2020-10-21

**Authors:** Yang Gu, Shulan Zhang

**Affiliations:** Department of Obstetrics and Gynecology, Shengjing Hospital of China Medical University, No. 36, Sanhao Street, Heping District, Shenyang, 110004 Liaoning P. R. China

**Keywords:** High-throughput sequencing, MicroRNA sequencing, MicroRNA-7-5p, TGFβ2, Ovarian cancer, Metastasis, In vitro validation

## Abstract

**Background:**

Ovarian cancer (OC) is a common gynecological cancer and characterized by high metastatic potential. MicroRNAs (miRNAs, miRs) have the promise to be harnessed as prognostic and therapeutic biomarkers for OC. Herein, we sought to identify differentially expressed miRNAs and mRNAs in metastatic OC, and to validate them with functional experiments.

**Methods:**

Differentially expressed miRNAs and mRNAs were screened from six pairs of primary OC tissues and metastatic tissues using a miRStar™ Human Cancer Focus miRNA and Target mRNA PCR Array. Then, gene expression profiling results were verified by reverse transcription quantitative polymerase chain reaction (RT-qPCR) and western blot assays. The binding affinity between miR-7-5p and TGFβ2 was validated by dual-luciferase reporter assay. Expression of miR-7-5p and TGFβ2 was manipulated to assess their roles in malignant phenotypes of highly metastatic HO-8910PM cells.

**Results:**

MiRNA profiling and sequencing identified 12 miRNAs and 10 mRNAs that were differentially expressed in metastatic tissues. Gene ontology and Pathway analyses determined that 3 differentially expressed mRNAs (ITGB3, TGFβ2 and TNC) were related to OC metastasis. The results of RT-qPCR confirmed that the decrease of miR-7-5p was most significant in OC metastasis, while TGFβ2 was up-regulated in OC metastasis. Moreover, miR-7-5p targeted and negatively regulated TGFβ2. MiR-7-5p overexpression accelerated HO-8910PM cell viability and invasion, and TGFβ2 overexpression reversed the results. Meanwhile, simultaneous miR-7-5p and TGFβ2 overexpression rescued the cell activities.

**Conclusions:**

This study characterizes differentially expressed miRNAs and mRNAs in metastatic OC, where miR-7-5p and its downstream target were most closely associated with metastatic OC. Overexpression of miR-7-5p targets and inhibits TGFβ2 expression, thereby inhibiting the growth and metastasis of OC.

## Background

Ovarian cancer (OC) is the deadliest gynecological cancer, due to the absence of symptoms at the early stage [1, 2]. The non-specific symptoms of OC contribute to a delayed diagnosis at advanced stages of cancer, which causes poor 5-year overall survival rate that is about 30–40% [3]. Moreover, the widespread and distant metastases are observed in 59% OC patients, which are clearly correlated with unfavorable prognosis of OC patients [4]. Therefore, it is pivotal to unveil invasive and metastatic mechanisms of OC. Of note, accurate prognostic strategies with the assistance of biomarker targets would aid in the diagnosis and prognosis of OC patients [5]. However, the understanding of mechanisms behind biomarkers and signaling pathways controlling invasive and metastatic potential of OC should been thoroughly probed [6].

MicroRNAs (miRNAs), small RNA molecules [21–23 nucleotides (nt)], function as potent modulators of gene expression through mRNA translation blockade or RNA interference [7]. MiRNAs have become a hotspot of tumor researches over the recent years. Interestingly, the differential expression of miRNAs has been reported in OC. For example, in a previous study conducted by Chong et al*.* [8] differential miRNA expression profiles showed that miR-551b, miR-19b, miR-196b and miR-3198 were upregulated and miR-8084, miR-3201, miR-3613 and miR-7515 were downregulated in OC. Besides, another study revealed that there were 17 differentially expressed miRNAs in metastatic OC, among which miR-21, miR-150, and miR-146a were upregulated in metastatic OC tumors [9].

Moreover, the dysregulated miRNAs may act as novel oncogenes or anti-oncogenes in OC by regulating target genes [10]. For instance, miR-7 was found to block the EGFR/ERK pathway to decrease OC cell invasion [11]. Stated thus, a hypothesis can be made that miRNAs may participate in OC metastasis via modulating their target genes. Notably, emerging evidence has demonstrated the importance of the miRNA-mRNA interplay in the pathogenesis and metastasis of OC [12, 13]. Therefore, differentially expressed miRNAs and mRNAs were screened from primary and metastatic tissues of OC using high-throughput sequencing technique. We found 12 miRNAs and 10 mRNAs that were differentially expressed in and related to metastatic potential of OC. The expression profiling results were validated by functional experimental validations.

## Methods

### Study participants

All 31 pairs of metastatic tissues (lymph node metastasis tissue) and primary tissues of OC were harvested from patients receiving surgery at Shengjing Hospital of China Medical University from 2014 to 2016. Of these, 6 pairs were used for expression profiling experiments, and 25 pairs were employed for RT-qPCR validation. Tissue samples resected during surgery were immediately placed into 1.5-mL centrifuge tubes with RNase-removal high-pressure treatment, and rapidly placed in liquid nitrogen for quick freezing, and then transferred to a − 80 ˚C cryogenic freezer for preservation. All patients were diagnosed with ovarian serous cystadenocarcinoma by postoperative paraffin pathology, and their clinical staging was made according to the International Federation of Gynecology and Obstetrics (FIGO) staging system for OC (2009). None of the patients had undergone preoperative radiotherapy, chemotherapy or other special treatments. Patients who provided tissue specimens for expression profiling experiments were aged 48–64 years with the mean age of 57.5 years, and those providing samples for RT-qPCR validation were aged 38–80 years with the mean age of 57 years. Data and tissues were harvested upon receiving the informed consent of patients and approval by the ethics committee of Shengjing Hospital of China Medical University (Approval number: 2016PS040K).

### RNA extraction from tissues

A Trizol Kit (Invitrogen Inc., Carlsbad, CA, USA) was applied for isolation of total RNA from metastatic and primary tissues of OC. RNA purity was estimated by adopting a NanoDrop^®^ ND-1000 spectrophotometer (Thermo Fisher Scientific, Waltham, MA, USA), and assessed by the ratio of optical density (OD) 260 nm/280 nm. A ratio between 1.8 and 2.0 signified the sample to be highly pure.

### MiRNA profiling and sequencing

Six pairs of metastatic and primary tissues of OC were obtained from Shengjing Hospital of China Medical University. Total RNA was extracted from tissues using a Trizol Kit. cDNA was synthesized, purified, and hybridized based on the instructions of an Arraystar miRNA First-Strand cDNA Synthesis Kit (Cat# AS-MR-004), which was fully compatible with an miRStar™ Human Cancer Focus miRNA and Target mRNA PCR Array (Arraystar, Rockville, MD, USA). After standardization of the original data from fluorescence quantitative PCR, fold changes in miRNAs and mRNAs were calculated based on a comparison between metastatic and primary tissues of OC. A fold change > 1.5 and *p* < 0.05 were considered as a noteworthy up-regulation, while fold change < − 1.5 and* p* < 0.05 were considered as a striking down-regulation.

### Gene ontology (GO) functional enrichment and pathway analyses

GO analysis and Kyoto Encyclopedia of Genes and Genomes (KEGG) enrichment analysis were employed to analyze differentially expressed mRNAs in OC based on a David database (https://david.ncifcrf.gov/). GO analysis provided annotations and function classifications for differentially expressed mRNAs by analyzing the classifications of Biological Processes, Cellular Components and Molecular Function. Pathway analysis was based on KEGG results combined with Fisher's exact test and a *t* test. A *p* < 0.05 was regarded statistically distinct.

### The miRNA-mRNA network analysis

Using a miRStar™ Human Cancer Focus miRNA and Target mRNA PCR Array expression profiling, we carried out the screening of 6 pairs of metastatic and primary tissues of OC in 184 cancer-related miRNAs and 178 target mRNAs. The miRNA-mRNA networks with a potential targeting relationship were screened out from miRNA microarray analysis and then plotted with the assistance of online tools targetscan (https://www.targetscan.org/vert_71/) and miranda (https://34.236.212.39/microrna/home.do).

### RT-qPCR

The metastatic and primary tissues of OC frozen at − 80 °C were collected. TRIzol reagent (Invitrogen) was applied for total RNA isolation from tissues and cells. A mixture (14.5 µL) of 2.0 µg RNA, 1 µL Oligo, 1.6 µL deoxy-ribonucleoside triphosphate Mix and H_2_O was incubated at 65 °C in water for 5 min and then put on ice for 2 min. After centrifugation, the mixture was mixed with reverse transcription reaction liquid, and placed at 37 °C for 1 min. The mRNA was reversely transcribed into cDNA, and the samples were then temporarily placed on ice. All cDNA template samples were prepared in a PCR amplification system. The fluorescence value of SYBR Green was detected. PCR primer sequences (Table 1) were designed and synthesized by Sangon Biotech (Shanghai, China). The relative expression of mRNAs or miRNAs, normalized to β-actin or U6, was calculated using 2^−ΔΔCt^ method.

**Table 1:** Primer sequences for RT-qPCR

Genes	Primer sequences
*miR-141-3p*	Forward: 5′-GGGGTAACACTGTCTGGTAA-3′
	Reverse: 5′-TGCGTGTCGTGGAGTC-3′
*miR-187-5p*	Forward: 5′-GGGGAGGCTACAACACAGGA-3′
	Reverse: 5′-TGCGTGTCGTGGAGTC-3′
*miR-7-5p*	Forward: 5′-GGGGGTGGAAGACTAGTGATTT-3′
	Reverse: 5′-GTGCGTGTCGTGGAGTCG-3′
*miR-584-5p*	Forward: 5′-TGGGTTATGGTTTGCCTGG-3′
	Reverse: 5′-GTGCGTGTCGTGGAGTCG-3′
*miR-200a-3p*	Forward: 5′-TAACACTGTCTGGTAACGATGT-3′
	Reverse: 5′-CATCTTACCGGACAGTGCTGGA-3′
*miR-200b-3p*	Forward: 5′-GCTGCTGAATTCCATCTAATTTCCAAAAG-3′
	Reverse: 5′-TATTATGGATCCGCCCCCAGGGCAATGGG-3′
*miR-9-3p*	Forward: 5′-GAGGCCCGTTTCTCTCTTTG-3′
	Reverse: 5′-AGCTTTATGACGGCTCTGTG-3′
*TGFβ2*	Forward: 5′-CTGCTAATGTTATTGCCCTCCTAC-3′
	Reverse: 5′-CCTGTAACAACGCATCTCATATT-3′
*TNC*	Forward: 5′ GTGACAGAAGTGACGGAAGAGAC3′
	Reverse: 5′ GATGGCAAATACACGGATAAAGT3′
*HOXB5*	Forward: 5′-GGCGCATGAAGTGGAA-GAAGGACA-3′
	Reverse: 5′-GAGAGGGAGCCACAGGAAGACC-3′
*U6*	Forward: 5′-GCTTCGGCAGCACATATACTAAAAT-3′
	Reverse: 5′-CGCTTCACGAATTTGCGTGTCAT-3′
*β-actin*	Forward: 5′-GTGGCCGAGGACTTTGATTG-3′
	Forward: 5′-CCTGTAACAACGCATCTCATATT-3′

*miR* microRNA, *TGFβ2* transforming growth factor beta 2, *TNC* Tenascin-C, *HOXB5* homeobox B5, *RT-qPCR* reverse transcription quantitative polymerase chain reaction

### Western blots

Tissues were immersed in radioimmunoprecipitation assay lysis buffer (P0013B; Beyotime, Shanghai, China) containing phenylmethyl sulfonylfluoride to extract total protein. The samples were allowed to stand on ice for 30 min, followed by 20-min centrifugation at 12,000×*g*. The supernatant was collected and transferred to an EP tube to determine the total protein concentration according to the instructions of a bicinchoninic acid kit (KC-430, Shanghai Kangchen Biological Engineering Co., Ltd., Shanghai, China). The samples were separated at 100 V for 1.5 h, and electroblotted onto a polyvinylidene fluoride membrane using a sandwich method at 80 V for 1.5 h. After 1-h blocking in 5% bovine serum albumin-Tris-buffered saline (TBST), the membrane was incubated with diluted (1:1000) rabbit anti-human TGFβ2 primary antibody overnight at 4 °C. Afterwards, the membrane was then re-probed with goat anti-rabbit secondary antibody (1:5000; Shanghai Kangchen Biological Engineering Co., Ltd., Shanghai, China) complexed to horseradish peroxidase at RT for 1 h. After rinsing with TBST, the membrane was soaked in electrogenerated chemiluminescence (P0018, Beyotime) for 1 min, developed, photographed and recorded. β-actin (1:10,000; Cell Signaling Technology, Danvers, MA, USA) served as an endogenous control for TGFβ2. A Gel-Pro Analyzer 4.0 program (Media Cybernetics, Silver Spring, MD, USA) was adopted for gray value analysis. The gray value ratio of each target protein band to the endogenous control band was calculated as the relative expression of each protein. Experiments were independently conducted three times.

### Dual-luciferase reporter assay

Binding sites for miR-7-5p and TGFβ2-3′untranslated region (UTR) were predicted using Genebank and Targetscan combined with expression profiling analysis results. TGFβ2-3′-UTR wild type (WT), TGFβ2-3′-UTR mutant type (MUT) luciferase plasmid and miR-7-5p mimic/negative control (NC) were constructed by Shanghai GeneChem Co., Ltd (Shanghai, China). After plasmid transfection, commonly used tool HEK293T cells in the logarithmic growth phase were prepared into a suspension that was then cultured in a 24-well plate so as to achieve 85% confluence at 24 h. Four groups were used in the experiment (NC mimic + TGFβ2-WT, miR-7-5p mimic + TGFβ2-WT, NC mimic + TGFβ2-MUT, and miR-7-5p mimic + TGFβ2-MUT). After 48-h transfection, the cells were lysed to obtain the supernatant that was centrifuged for 3–5 min. The luciferase activity of TGFβ2 3′UTR in response to miR-7-5p mimic was detected by utilizing a Dual-Luciferase^®^ Reporter Assay System (E2910; Promega, Fitchburg, WI, USA). The luminescence intensity was measured by GloMax^®^20/20 luminometer (Promega, Madison, WI, USA).

### Cell treatment and grouping

The cells used for the cell phenotype detection were the human highly metastatic OC cell line HO-8910PM, which has also been previously reported to establish a highly metastatic animal model [14, 15]. Besides, the human normal ovarian cell line HUM-CELL-0088 was selected as a control. Both cell lines were purchased from Shanghai Institute of Biochemistry and Cell Biology (Shanghai, China). HEK293T cells (used for dual-luciferase reporter assay) were cultured in Dulbecco minimum essential medium (DMEM; 12800017; Gibco, Carlsbad, CA, USA) containing 10% fetal bovine serum (FBS; FBS500-S; Ausgenex, Brisbane, Australia) in an incubator (BB15; Thermo Fisher Scientific, Waltham, MA, USA) with saturated humidity and 5% CO_2_ at 37 °C. HO-8910-PM cells and HUM-CELL-0088 cells were cultured in RPMI-1640 medium (Gibco-BRL, Life Technologies, Gaithersburg, MD, USA) containing 10% FBS in an incubator with saturated humidity and 5% CO_2_ at 37 °C. The medium was renewed every 24 h, and subculture was performed every 72 h. After removal of medium, cells were treated with 0.25% trypsin for 3 min, and then the reaction was ended by adding RPMI-1640 medium containing 10% FBS. Afterwards, cells were pipetted into single cell suspension. Then HO-8910-PM cells were transduced with lentiviral vectors containing miR-7-5p mimic, NC mimic, miR-7-5p inhibitor, NC inhibitor, TGFβ2 overexpression plasmid (oe-TGFβ2) or oe-NC. Lentiviruses were purchased from Shanghai Genechem Co., Ltd. (Shanghai, China). The negative control and miR-7-5p mimic and inhibitor were obtained from GenePharma (Shanghai, China). The sequence of the negative control, miR-7-5p mimic and inhibitor were provided in Additional file 1: Table S1. Cells in logarithmic growth phase were treated with trypsin to prepare into 3 × 10^4^ cells/mL cell suspension. Cell suspension (2 mL) was seeded into 6-well plates for further culture. The infection medium (Eni.S + polybrene) was renewed, and the cell density was 20%/well at the time of infection. The optimal amount (infection MOI = 10) was added for infection. Twelve hours after infection, the conventional medium was renewed to continue culture cells.

### Cell counting kit (CCK-8) assay

The 80% confluent cells were dispersed into single cell suspension with the reaction of 0.25% trypsin. After counting, the cells were separately seeded in a 96-well plate with 100 μL (2 × 10^4^ cells/mL) per well, and set with 6 duplicated wells. Cells were cultured in the incubator, and 10 μL CCK-8 solutions (Sigma-Aldrich, St. Louis, MO, USA) were supplemented to each well after culture for 24 h, 48 h, 72 h, and 96 h. After further 2-h culture, OD value at 450 nm was measured using an enzyme-linked immunometric meter. Cell viability curve was drawn with time as X-axis and OD value as Y-axis.

### Transwell assay

After transfection, cells were suspended in 0.25% trypsin (C0205, Beyotime), followed by centrifugation at 400 rpm for 5 min with the supernatant discarded. Subsequently, cells were resuspended in RPMI-1640 medium. Cell density was adjusted to 10^5^ cells/mL. Before the experiment, Transwell chambers (3413, Millipore, Massachusetts, USA) were coated with Matrigel. The 100 μL cell suspension was supplemented into the 24-well apical chamber (10^5^ cells/well). RPMI-1640 medium (600 μL) containing 30% FBS was added into the basolateral chamber. Each group was set 3 duplicated wells. After 48-h culture, cells not invading through Matrigel were discarded using cotton swabs, and cells invading through Matrigel were stained with 0.1% Giemsa (32884, Sigma-Aldrich) for 10 min. The number of cells invading through Matrigel was counted in five random fields under the microscope. The number of cells invading through Matrigel was considered as an indicator of the invasion ability.

### Scratch test

Even horizontal linear scratches were made in the back of 6-well plate by a marker pen with 3 scratches in each well. Six pairs of cells at logarithmic growth phase were seeded in the culture plate (5 × 10^5^ cells/well). Following 24-h culture, when cells settled at the bottom of the well, 200 μL pipette tips was adopted to make even vertical linear scratches in the back of the plate with 3 scratches in each well. Afterwards, cell incubation was conducted in RPMI-1640 medium with 5% CO_2_ at 37 °C. The migration distance was observed and photographed under inverted microscope at 0 h and 24 h after scratch. Three replicates were made in each group. The number of cells passing through scratches was considered as an indicator of the migration ability.

### Flow cytometry

Overnight cell fixative was conducted in 70% chilled ethanol at 4 °C. After centrifugation at 800×*g* and 4 °C with the removal of supernatant, cells were washed with phosphate buffer saline (PBS) containing 1% FBS. Cells were resuspended with 400 μL binding buffer, followed by 30-min incubation with 50 μL RNAase at 37 °C. After that, cells were added with 50 μL propidium iodide (PI) (50 mg/L, Sigma-Aldrich) for 30-min incubation in dark. Flow cytometry was used to detect cell cycle.

### Statistical analysis

All data were summarized as mean ± standard deviation and analyzed using SPSS 21.0 statistical software (IBM Corp., Armonk, New York, USA). Data between two groups were compared using unpaired *t-*test. Statistical analysis in relation to time-based measurements within each group was realized using repeated measures analysis of variance (ANOVA), followed by a Bonferroni’s post-hoc test. Pearson correlation coefficient was adopted for correlation analyses. Significance was defined as *p* < 0.05.

## Results

### Differentially expressed miRNAs and mRNAs with metastatic OC

**Fig. 1:**
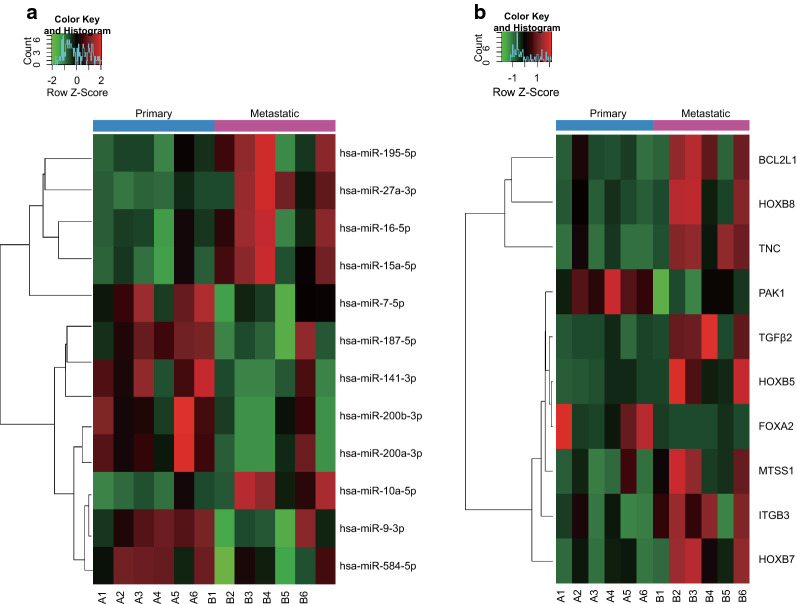
Differentially expressed miRNAs and mRNAs with metastatic OC. **a** Heat map of OC-related differentially expressed miRNAs in metastatic tissues and primary tissues of OC. **b** Heat map of OC-related differentially expressed mRNAs in metastatic tissues and primary tissues of OC. The color red represents up-regulated expression and the color green represents down-regulated expression

Differentially expressed miRNAs and mRNAs in metastatic OC were screened using miRStar™ Human Cancer Focus miRNA and Target mRNA PCR Array analysis. As shown in Table 2, 12 differentially expressed miRNAs were screened out from metastatic and primary tissues of OC (Fig. 1a): (in sequence of absolute fold change) miR-200a-3p, miR-141-3p, miR-200b-3p, miR-10a-5p, miR-15a-5p, miR-187-5p, miR-16-5p, miR-9-3p, miR-195-5p, miR-7-5p, miR-584-5p and miR-27a-3p (*p* < 0.05). The expression of miR-10a-5p, miR-15a-5p, miR-16-5p, miR-195-5p and miR-27a-3p in metastatic tissues from OC was increased, as compared with primary OC tissues (*p* < 0.05, fold change > 1.5), while that of miR-141-3p, miR-7-5p, miR-187-5p, miR-200a-3p, miR-200b-3p, miR-584-5p and miR-9-3p was lower than that in primary OC tissues (*p* < 0.05, fold change < − 1.5). As shown in Table 3, a total of 10 differentially expressed target mRNAs were identified. Relative to primary OC tissues, the gene expression of BCL2L1, TGFβ2, TNC, HOXB5, HOXB7, HOXB8, ITGB3 and MTSS1 in metastatic tissues from OC was substantially up-regulated (*p* < 0.05, fold change > 2; Fig. 1b), while that of PAK1 and FOXA2 was down-regulated (*p* < 0.05, fold change < − 2).

**Table 2:** Differentially expressed miRNAs in metastatic tissue compared with primary OC tissue

Genes	Metastatic (2^−ΔCt^)	Primary (2^−ΔCt^)	*P* value	Fold change
*miR-10a-5p*	9.10E−03	3.20E−03	0.014557	2.85
*miR-15a-5p*	2.90E−01	1.30E−01	0.008198	2.24
*miR-16-5p*	7.30E−01	4.00E−01	0.042838	1.84
*miR-195-5p*	1.00E+00	6.10E−01	0.047072	1.66
*miR-584-5p*	5.40E−02	8.50E−02	0.032832	− 1.58
*miR-27a-3p*	7.60E−01	4.90E−01	0.003931	1.54
*miR-141-3p*	3.10E−02	1.70E−01	0.013728	− 5.64
*miR-187-5p*	9.50E−02	1.80E−01	0.031732	− 1.90
*miR-200a-3p*	5.60E−03	3.40E−02	0.023611	− 5.99
*miR-200b-3p*	1.20E−02	5.20E−02	0.022363	− 4.53
*miR-7-5p*	2.20E−01	3.60E−01	0.020499	− 1.65
*miR-9-3p*	1.90E−03	3.40E−03	0.040069	− 1.76

*miR* microRNA, *OC* ovarian cancer

**Table 3:** Differentially expressed mRNAs in metastatic tissue compared with primary OC tissue

Genes	Metastatic (2^−ΔCt^)	Primary (2^−ΔCt^)	*P* value	Fold change
*BCL2L1*	4.70E−02	2.30E−02	0.047085	2.07
*TGFβ2*	2.00E−03	3.50E−04	0.017231	5.68
*TNC*	5.20E−02	2.50E−03	0.006661	20.7
*HOXB5*	6.10E−04	1.00E−04	0.035099	6.11
*HOXB7*	1.20E−02	2.50E−03	0.033375	4.9
*HOXB8*	4.10E−02	5.70E−03	0.046631	7.19
*ITGB3*	5.60E−03	1.40E−03	0.021938	3.9
*MTSS1*	5.10E−03	1.00E−03	0.034451	5.08
*FOXA2*	1.70E−05	1.10E−04	0.034065	− 6.48
*PAK1*	2.60E−03	5.40E−03	0.006704	− 2.04

*BCL2L1* B-cell CLL/lymphoma 2-like 1, *TGFβ2* transforming growth factor beta 2, *TNC* tenascin-C, *HOX* homeobox, *ITGB3* integrin beta-3, *MTSS1* metastasis suppressor 1, *FOXA2* forkhead box A2, *PAK1* p21-activated kinase, *OC* ovarian cancer

### GO functional enrichment and Pathway analyses for the identified differentially expressed mRNAs

**Fig. 2:**
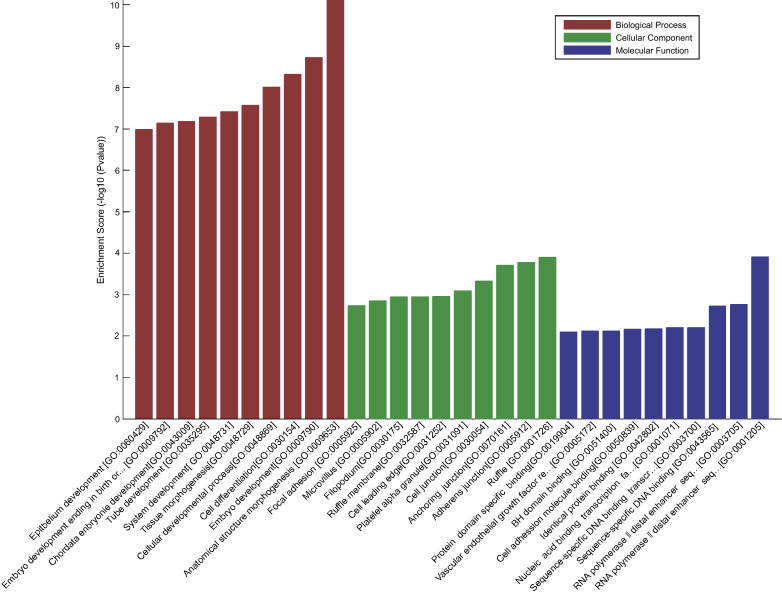
GO functional enrichment analysis for 10 differentially expressed mRNAs. The DAVID database (https://david.ncifcrf.gov/; version: 6.8) was used to perform GO analyses. The top 10 items of these enrichment analyses are conducted by using the “ggplot2” package in R software. *P* < 0.05 was considered statistically significant; *DAVID* database for annotation, visualization and integrated discovery, *GO* gene ontology. The top 10 items of GO categories including biological process (BP), cellular component (CC), and molecular function (MF) were then sorted and displayed in the form of maps

**Fig. 3:**
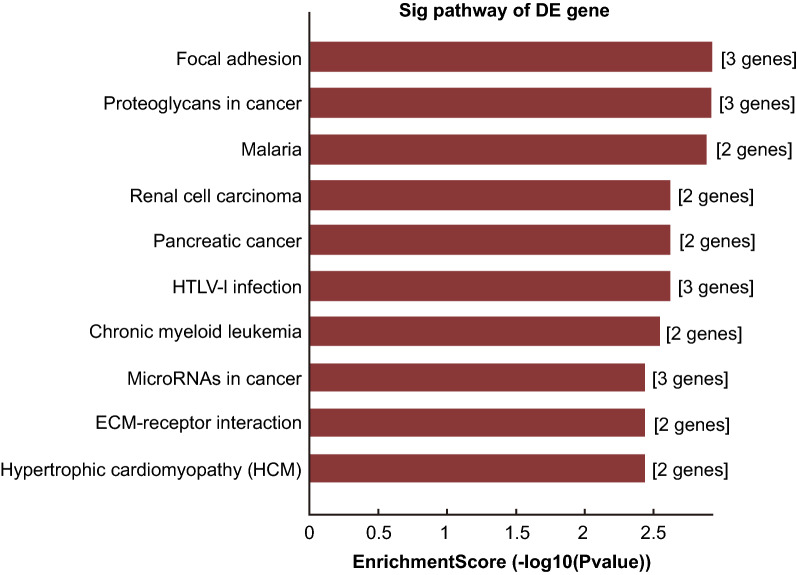
Pathway analysis based on KEGG method for 10 differentially expressed mRNAs. The DAVID database (https://david.ncifcrf.gov/; version: 6.8) was used to perform KEGG analyses. The top 10 items of these enrichment analyses are conducted by using the “ggplot2” package in R software. *P* < 0.05 was considered statistically significant. KEGG: Kyoto Encyclopedia of Genes and Genomes; DAVID: database for annotation, visualization and integrated discovery

To determine which mRNAs were involved in the regulatory effect of miRNAs in metastatic OC, GO analysis (Fig. 2) was performed to investigate the function of 10 differentially expressed mRNAs. For Biological Processes, 10 differentially expressed mRNAs mostly participated in anatomical structure morphogenesis, embryo development, cell differentiation and cellular developmental processes (*p* < 0.001, Enrichment scores > 8; Table 4). For cellular components, the differentially expressed mRNAs mostly participated in ruffle, adherens junction, anchoring junction and cell junction (*p* < 0.001, Enrichment scores > 3.3; Table 5). For molecular functions, the differentially expressed mRNAs mostly participated in RNA polymerase II distal enhancer sequence-specific DNA binding transcription factor activity involved in positive regulation of transcription (*p* < 0.001, Enrichment scores > 3; Table 6). Furthermore, the Pathway analysis based on KEGG method (Fig. 3) indicated that, among the 10 differentially expressed mRNAs, ITGB3, TGFβ2 and TNC were linked to the pathway of MicroRNAs in Cancer.

**Table 4:** Nodes in biological processes found by gene ontology

GO.ID	Term	Count	*P* value	Enrichment score
GO:0009653	Anatomical structure morphogenesis	12	7.67E−11	10.11505
GO:0009790	Embryo development	9	1.88E−09	8.72576
GO:0030154	Cell differentiation	12	4.76E−09	8.322078
GO:0048869	Cellular developmental process	12	9.76E−09	8.010594
GO:0048729	Tissue morphogenesis	7	2.67E−08	7.573257
GO:0048731	System development	12	3.86E−08	7.412918
GO:0035295	Tube development	7	5.16E−08	7.287302
GO:0043009	Chordate embryonic development	7	6.66E−08	7.176411
GO:0009792	Embryo development ending in birth or egg hatching	7	7.21E−08	7.142
GO:0060429	Epithelium development	8	1.02E−07	6.989655

**Table 5:** Nodes in cellular components found by gene ontology

GO.ID	Term	Count	*P* value	Enrichment score
GO:0001726	Ruffle	3	0.000125	3.901568
GO:0005912	Adherens junction	4	0.000167	3.777312
GO:0070161	anchoring junction	4	0.000195	3.709575
GO:0030054	Cell junction	5	0.000474	3.324369
GO:0031091	Platelet alpha granule	2	0.000817	3.087852
GO:0031252	Cell leading edge	3	0.001105	2.956574
GO:0030175	Filopodium	2	0.00113	2.946761
GO:0032587	Ruffle membrane	2	0.00113	2.946761
GO:0005902	Microvillus	2	0.001389	2.857181
GO:0005925	Focal adhesion	3	0.001841	2.73493

**Table 6:** Nodes in molecular functions found by gene ontology

GO.ID	Term	Count	*P* value	Enrichment score
GO:0001205	RNA polymerase II distal enhancer sequence-specific DNA binding transcription factor activity involved in positive regulation of transcription	2	0.000122	3.911881
GO:0003705	RNA polymerase II distal enhancer sequence-specific DNA binding transcription factor activity	2	0.001717	2.765282
GO:0043565	Sequence-specific DNA binding	4	0.001875	2.727084
GO:0003700	Sequence-specific DNA binding transcription factor activity	4	0.006255	2.203762
GO:0001071	Nucleic acid binding transcription factor activity	4	0.006277	2.202281
GO:0042802	Identical protein binding	4	0.006603	2.180266
GO:0050839	Cell adhesion molecule binding	2	0.006752	2.170542
GO:0005172	Vascular endothelial growth factor receptor binding	1	0.007614	2.118391
GO:0051400	BH domain binding	1	0.007614	2.118391
GO:0019904	Protein domain specific binding	3	0.008101	2.091458

### The down-regulation of miR-7-5p is the most significant in metastatic tissue of OC

**Fig. 4:**
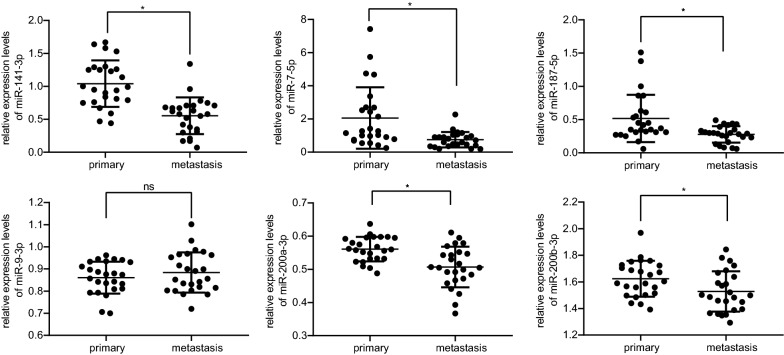
The expression of the 6 identified down-regulated miRNAs (miR-7-5p, miR-187-5p, miR-141-3p, miR-200a-3p, miR-200b-3p, and miR-9-3p) in metastatic tissues and primary tissues of OC as detected by RT-qPCR. Relative expression of miRNA was measurement data and analyzed by unpaired *t* test. N = 25. ^*^p < 0.05 compared with primary OC tissues. *ns* no significance

Next, we moved to assess expression of the screened differentially expressed miRNAs as mentioned above, so as to determine the research focus for this present study. As illustrated in Fig. 4, when compared with primary OC tissues, there were 5 miRNAs miR-7-5p, miR-141-3p, miR-187-5p, miR-200a-3p and miR-200b-3p were appreciably decreased in metastatic tissues, where miR-7-5p expression presented with the most significant decrease. Thus, miR-7-5p was selected as a research focus for following experiments.

### The up-regulation of TGFβ2 is the most significant in metastatic tissue of OC

**Fig. 5:**
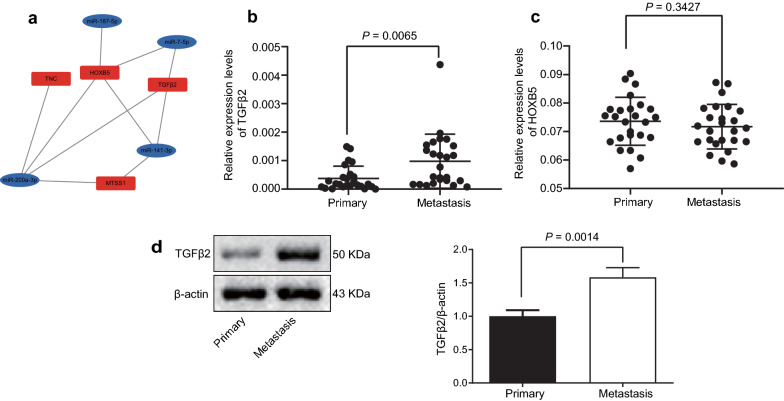
The up-regulation of TGFβ2 is the most significant in metastatic tissue of OC. **a** Differentially expressed miRNA-mRNA network. **b**, **c** The mRNA expression of TGFβ2 (**b**) and HOXB5 (**c**) in metastatic tissues and primary tissues of OC as measured by RT-qPCR. **d** Western blots and quantitation of protein level of TGFβ2 in metastatic tissues and primary tissues of OC. Relative expression of miRNA and protein was measurement data and analyzed by unpaired *t* test. N = 25. ^*^p < 0.05 compared with primary OC tissues

Further, we proceeded to probe the downstream target genes of aforementioned miR-7-5p. A differentially expressed miRNA-mRNA network (Fig. 5a) was plotted, where we found that the target genes of miR-7-5p were TGFβ2 and HOXB5. Therefore, mRNA expression of TGFβ2 and HOXB5 was measured by RT-qPCR (Fig. 5b, c). Relative to the primary OC tissues, expression of TGFβ2 mRNA was enhanced in metastatic tissues (*p* < 0.05), while no significant difference was witnessed in the slightly up-regulated expression of HOXB5 mRNA (*p* > 0.05). This may be implicated in factors, such as sample size and ethnic differences. In addition, western blot assay (Fig. 5d) further confirmed that the protein level of TGFβ2 was elevated in metastatic tissues versus that in primary OC tissues (*p* = 0.0014). Accordingly, we selected miR-7-5p and its downstream target TGFβ2 for further investigations.

### miR-7-5p targets and inhibits TGFβ2

**Fig. 6:**
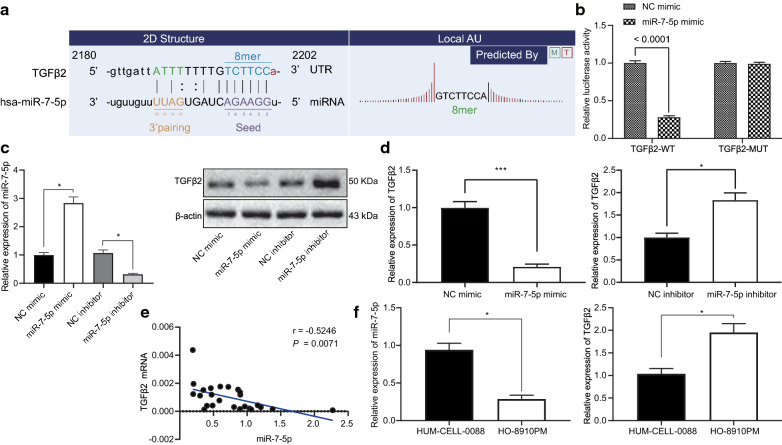
MiR-7-5p targets and inhibits TGFβ2 expression. **a** Predicted binding sites between miR-7-5p on TGFβ2 3′-UTR. **b** Dual luciferase reporter assay verifying the binding of miR-7-5p to TGFβ2 in commonly used tool HEK293T cells. **c** RT-qPCR detection (left) of relative expression of miR-7-5p and Western blots (right) of protein level of TGFβ2 in highly metastatic HO-8910PM cells in the presence of miR-7-5p mimic or inhibitor. **d** The mRNA expression of TGFβ2 in highly metastatic HO-8910PM cells in the presence of miR-7-5p mimic or inhibitor as measured by RT-qPCR. **e** The negative correlation between miR-7-5p and TGFβ2. **f** The expression of miR-7-5p and TGFβ2 in normal ovarian cell line HUM-CELL-0088 and highly metastatic OC cell line HO-8910PM as measured by RT-qPCR. Relative expression of miRNA and protein was measurement data and analyzed by unpaired *t* test. *p < 0.05, **p < 0.01, ***p < 0.001 compared with NC mimic group or HUM-CELL-0088 cells. The cell experiment was repeated three times

We used the commonly used tool cell HEK293T cells to conduct a dual luciferase reporter gene assay to verify the targeting relationship between miR-7-5p and TGFβ2. It was found that the luciferase activity of TGFβ2-WT was reduced in response to miR-7-5p mimic transfection, while there was no significant difference in luciferase activity of TGFβ2-MUT (Fig. 6a, b), suggesting that miR-7-5p could target TGFβ2. Furthermore, we transfected miR-7-5p mimic or miR-7-5p inhibitor into highly metastatic OC cell line HO-8910PM to validate its regulatory effect on TGFβ2. As shown in Fig. 6c, we have also tested the transfection efficiency of miR-7-5p inhibitor and miR-7-5p mimic. The RT-qPCR detection presented that miR-7-5p expression was upregulated in the presence of miR-7-5p mimic and downregulated in the presence of miR-7-5p inhibitor. In addition, Western blot analysis manifested that protein levels of TGFβ2 was diminished in response to miR-7-5p mimic and elevated in response to miR-7-5p inhibitor in highly metastatic HO-8910PM cells (Fig. 6c, d). As shown in Fig. 6c, we have also tested the transfection efficiency of miR-7-5p inhibitor and miR-7-5p mimic. Besides, miR-7-5p expression was inversely correlated with TGFβ2 expression in metastatic tissues from OC (Fig. 6e).

Additionally, we also examined the expression of miR-7-5p and TGFβ2 in normal ovarian cell line HUM-CELL-0088 and highly metastatic OC cell line HO-8910PM. The RT-qPCR results (Fig. 6f) showed that, compared with normal ovarian cells, miR-7-5p was poorly expressed in highly metastatic OC cells, while TGFβ2 was highly expressed in highly metastatic OC cells. In conclusion, miR-7-5p directly bound to TGFβ2 3′UTR and negatively regulated its expression.

### Overexpression of miR-7-5p inhibits OC cell viability, migration and invasion by targeting TGFβ2

**Fig. 7:**
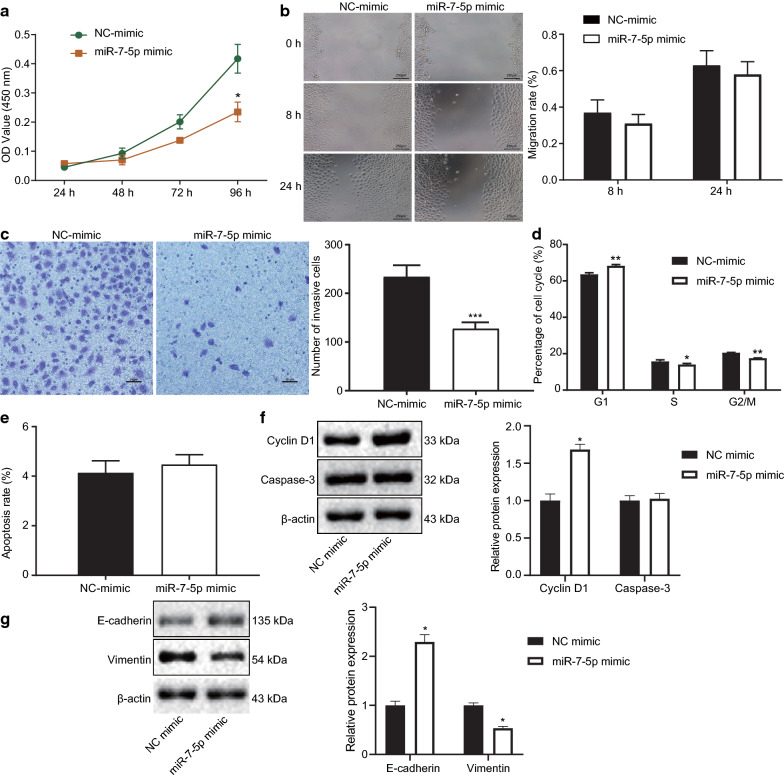
Overexpression of miR-7-5p inhibits OC cell viability, migration and invasion. **a** The viability of HO-8910PM cells after miR-7-5p overexpression as determined by CCK-8. **b** The migration of HO-8910PM cells after miR-7-5p overexpression as measured by Scratch test. **c** The invasion of HO-8910PM cells after miR-7-5p overexpression as determined by Transwell assay. **d** The cell cycle distribution of HO-8910PM cells after miR-7-5p overexpression as determined by flow cytometry. **e** The cell apoptosis rate of HO-8910PM cells after miR-7-5p overexpression as determined by Annexin V-APC staining. **f** Western blots and quantitation of protein level of cyclin D1 and caspase-3 in HO-8910PM cells after miR-7-5p overexpression. **g** Western blots and quantitation of protein level of epithelial–mesenchymal transition-related factors (E-cadherin and Vimentin) in HO-8910PM cells after miR-7-5p overexpression. Measurement data were presented as mean ± standard deviation, analyzed by unpaired *t* test. Statistical analysis in relation to time-based measurements within each group was realized using repeated measures ANOVA, followed by a Bonferroni’s post-hoc test. *p < 0.05, **p < 0.01, ***p < 0.001 compared with NC mimic group. The cell experiment was repeated three times

**Fig. 8:**
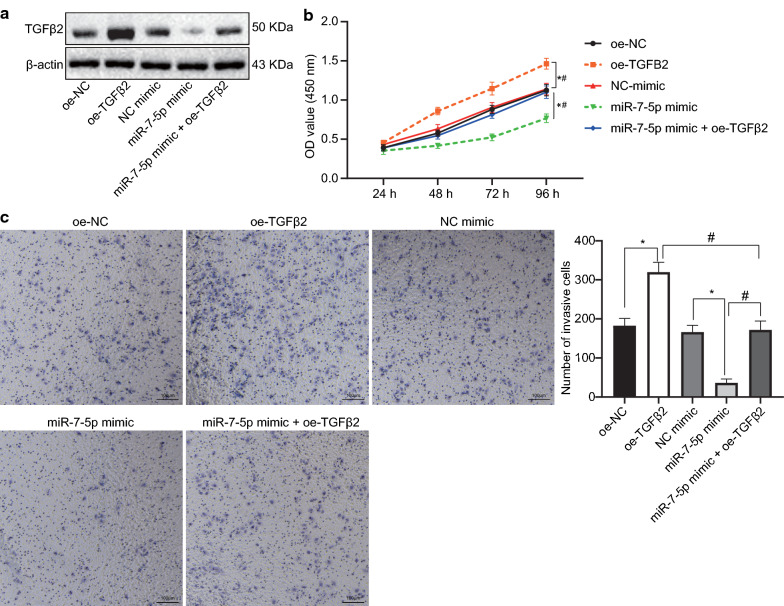
Overexpression of miR-7-5p inhibits OC cell viability and invasion by targeting TGFβ2. **a** miR-7-5p overexpression and TGFβ2 overexpression alone or in combination. **b** The viability of HO-8910PM cells in response to miR-7-5p overexpression and TGFβ2 overexpression alone or in combination as determined by CCK-8. **c** The invasion of HO-8910PM cells in response to miR-7-5p overexpression and TGFβ2 overexpression alone or in combination as determined by Transwell assay. *p < 0.05 compared with NC mimic group or oe-NC group. ^#^*p* < 0.05 compared with miR-7-5p mimic group or oe-TGFβ2 group. Data between two groups were analyzed using unpaired *t* test. The cell experiment was repeated three times

After determining the negative correlation between miR-7-5p and TGFβ2, we shifted to validate their roles functionally in malignant phenotypes of highly metastatic OC cell line HO-8910PM by using CCK-8 assay (Figs. 7a, 8b), Scratch test (Fig. 7b), Transwell invasion assay (Figs. 7c, 8c), flow cytometric detection (Fig. 7d), Annexin V-APC staining (Fig. 7e) and western blots (Fig. 7f). In the presence of miR-7-5p mimic, HO-8910PM cells presented with attenuated viability (Fig. 7a), migration (Fig. 7b) after 24 h (no significant difference observed at 8 h), and invasion (Fig. 7c). These results indicated that up-regulated miR-7-5p appreciably restricted the proliferative, migratory and invasive capacities of OC cells in vitro.

Moreover, the quantitative flow cytometric analysis (Fig. 7d) showed that the percentages of cells in G1 phase of the NC mimic group and the miR-7-5p mimic group were (63.62 ± 0.85)% and (68.32 ± 0.62)%, respectively; the percentages of cells in S phase of the NC mimic group and the miR-7-5p mimic group were (15.82 ± 0.79)% and (14.12 ± 0.52)%, respectively; and the percentages of cells in G2/M phase of the NC mimic group and the miR-7-5p mimic group were (20.56 ± 0.11)% and (17.57 ± 0.11)%, respectively. Accordingly, the miR-7-5p mimic treatment prolonged G1 phase (increased cell percentage) and shortened S and G2/M phases (reduced cell percentage). These data suggested that up-regulated miR-7-5p blocked OC cell cycle progression.

In terms of cell apoptosis detected by Annexin V-APC staining method (Fig. 7e), the apoptosis rate of HO-8910PM cells in the NC mimic group and miR-7-5p mimic group was lower than 5%, and there was no significant difference in the apoptosis rate between the two groups. Western blot assay further verified the effect of miR-7-5p mimic on cyclin D1 protein expression and caspase-3 activity in HO-8910PM cells. The results showed that overexpression of miR-7-5p up-regulated cyclin D1 protein expression, but had no effect on caspase-3 (Fig. 7f). Moreover, as shown in Fig. 7g, HO-8910PM cells transfected with miR-7-5p mimic presented with elevated expression of E-cadherin and lowered expression of Vimentin. This result indicated that overexpression of miR-7-5p suppressed epithelial–mesenchymal transition (EMT) of highly metastatic OC cell line HO-8910PM.

Additionally, we conducted rescue experiments (Fig. 8) on HO-8910PM cells overexpressing miR-7-5p and TGFβ2 simultaneously. Firstly, western blot analysis (Fig. 8a) suggested that expression of TGFβ2 protein was elevated in response to oe-TGFβ2 alone and reduced to miR-7-5p mimic alone, while the combined treatment of oe-TGFβ2 and miR-7-5p mimic rescued its expression. Furthermore, miR-7-5p mimic alone diminished viability and invasion of HO-8910PM cells, while TGFβ2 overexpression alone facilitated viability and invasion of HO-8910PM cells. However, simultaneous miR-7-5p and TGFβ2 overexpression rescued cell viability and invasion in response to miR-7-5p mimic or TGFβ2 overexpression alone (Fig. 8b, c).

## Discussion

MiRNAs confer pivotal roles in multiple cellular functions, such as proliferation, apoptosis and differentiation. However, aberrant expression of miRNAs appears in carcinogenesis and metastasis during cancer progression [16]. Mounting evidence highlights a crucial role of miRNAs in OC progression. For instance, alterations in miR-101, miR-206, miR-200a and miR-203 are involved with OC cell proliferation and invasion [17–19]. Therefore, this current study presented differentially expressed miRNAs and mRNAs related to metastatic OC and revealed a tumor-inhibiting role of miR-7-5p in the restriction of OC metastasis, which was associated with the regulation of TGFβ2.

Different types of cancer samples show varying gene expression patterns, and even the same miRNA often regulate various target genes in different types of cancers. In studies on OC, the application of a miRNA expression profiling technique is helpful in identifying differentially expressed miRNAs affecting malignant phenotypes and metastasis in OC in a more rapid and comprehensive manner. In the present study, we analyzed the miRNA expression profiles of primary OC tissues and metastatic tissues using a miRStar™ Human Cancer Focus miRNA and Target mRNA PCR Array expression profiling. With the application of a miRCURY LNA™ microRNA Array expression profiling technique, Liu et al*.* [20] identified 31 differentially expressed miRNAs from another two pairs of cisplatin-resistant OC cell lines, of which the expression of 21 miRNAs was up-regulated and that of 10 miRNAs was down-regulated. Moreover, Cheng et al*.* [21] found by using an Affymetrix miRNA 3.0 Array expression profiling that 37 miRNAs were differentially expressed in adult and juvenile granulosa cell tumors, and that these could be used as markers for the diagnosis and recurrence of ovarian granulosa cell tumors.

The high-throughput sequencing screening of this study found 12 differentially expressed miRNAs (miR-195-5p, miR-27a-3p, miR-16-5p, miR-15a-5p, miR-10a-5p, miR-584-5p, miR-7-5p, miR-187-5p, miR-141-3p, miR-200b-3p, miR-200a-3p and miR-9-3p) and 10 differentially expressed mRNAs (BCL2L1, TGFβ2, TNC, HOXB5, HOXB7, HOXB8, ITGB3, MTSS1, PAK1 and FOXA2) in OC. GO analysis and Pathway analysis finally determined that 3 differentially expressed mRNAs (ITGB3, TGFβ2 and TNC) were related to OC metastasis. The results of RT-qPCR confirmed that the decrease of miR-7-5p was most significant in OC metastases, while TGFβ2 was significantly up-regulated in OC metastases. Therefore, miR-7-5p and its downstream target gene TGFβ2 were selected as research focuses. A wide array of miRNAs have been reported to present differential expression in OC [22]. For instance, miR-187 has been found to exert a dual role in OC by regulating the disabled homolog-2 gene [23]. Gao et al*.* [24] also found that miR-141 acted as a potential diagnostic and prognostic biomarker for OC, which was consistent with our in silico prediction results. Of interest, underexpressed miR-7-5p has been documented in metastatic breast cancer [25] and invasive pancreatic cancer [26]. Another study also claimed that miR-7-5p deficiency was associated with recurrence in glioblastoma patients, and that its overexpression decreased glioblastoma cell stemness [27]. Furthermore, it was also elucidated that ectopic expression of miR-7 functioned as an anti-oncogene in OC by repressing cell invasion and proliferation [11].

Recently, a growing number of studies elucidated that miRNAs are implicated in the invasive and metastatic potential of tumors. One miRNA can regulate nearly 100 different mRNAs, and a single mRNA can bind to several different miRNAs, thus forming a miRNA-target gene network regulatory relationship [28, 29]. Of note, miRNAs can post-transcriptionally mediate a number of genes through the binding of specific sequences in target mRNA molecules [30]. TGFβ2 as a putative target of miR-7-5p was verified using a luciferase activity assay of the present study. Moreover, there was a negative correlation between miR-7-5p expression and TGFβ2 expression in OC tissues, and miR-7-5p targeted and negatively regulated TGFβ2. MiR-7-5p has been found to target and regulate target genes as SATB1 and PARP1 in some malignancies [31, 32]. For instance, Zhu et al*.* [33] demonstrated that miR-7-5p suppresses cell migratory and invasive potential by targeting SOX18 in pancreatic ductal adenocarcinoma.

In addition, through gain- and loss-of-function experiments in highly metastatic OC cells, our results revealed that miR-7-5p overexpression reduced OC cell viability and invasion, and blocked cell cycle entry by targeting TGFβ2. In addition, we found that OC metastatic tissues presented higher levels of TGFβ2 relative to primary OC tissues. TGFβ2 is an isoform of TGFβ in mammals [34] and is abnormally expressed in various cancers, such as human melanoma and hepatocellular carcinoma [35, 36]. Furthermore, TGFβ2 and the TGFβ pathway could interact with miRNAs, such as miR-187 and miR-141 identified in this study, to affect growth of tumors. Ectopic miR-187 expression could negate the contribution of TGFβ to aggressiveness in metastatic colorectal cancer [37]. Besides, the interplay of miR-141 and oncogenic TGFβ2 was found to orchestrate malignancy in gastric cancer [38]. Additionally, many researchers have also explored the role of TGFβ in OC. Cao et al. [39] reported that TGFβ-induced transglutaminase accelerated EMT and a cancer stem cell phenotype that consequently enhanced ovarian tumor metastasis. TGFβ induced EMT and a more invasive phenotype in epithelial OC cells in collaboration with the EGF pathway, indicating TGFβ may be a promising target candidate for the treatment of metastatic OC in future [40].

## Conclusion

In conclusion, our study suggested several differentially expressed miRNAs and mRNAs for metastatic OC, and uncovered the tumor-suppressive function of miR-7-5p in highly metastatic OC cells. miR-7-5p directly bound to TGFβ2 3′UTR to inhibit its expression, thus restricting the invasive and metastatic potential of OC. miR-7-5p may be harnessed as a potential target for preventive and therapeutic strategies against metastatic OC. Although bioinformatic analyses and in vitro findings shed new light on the mechanistic understanding of tumor-suppressive miR-7-5p and its target TGFβ2 in OC metastasis, the conclusions of this study need to be further confirmed in future studies based on in vivo experimental validations. Moreover, it is necessary to conduct further cohort studies with larger sample sizes to validate our results.

## Supplementary information

**Additional file 1: Table S1.** Sequences of the negative control, miR-7-5p mimic and inhibitor.

## Data Availability

The datasets generated and/or analyzed during the current study are available from the corresponding author on reasonable request.

## Abbreviations

OC: Ovarian cancer
miR or miRNA: MicroRNA
RT-qPCR: Reverse transcription quantitative polymerase chain reaction
OD: Optical density
KEGG: Kyoto Encyclopedia of Genes and Genomes
TBST: Tris-buffered saline
NC: Negative control
UTR: Untranslated region
WT: Wild type
FBS: Fetal bovine serum
CCK-8: Cell counting kit 8
PI: Propidium iodide
PBS: Phosphate buffer saline
