# Supplementary material for: High-throughput sequencing identification of differentially expressed microRNAs in metastatic ovarian cancer with experimental validations
Source: Cancer Cell Int. 2020 Oct 21;20:517. doi: 10.1186/s12935-020-01601-4 (PMC7579798; doi:10.1186/s12935-020-01601-4)
Supplement: Supplementary file 1 — Additional file 1: Table S1. Sequences of the negative control, miR-7-5p mimic and inhibitor. [file 12935_2020_1601_MOESM1_ESM.docx]

**Table S1** Sequences of the negative control, miR-7-5p mimic and inhibitor.

| Name | Sequence |
| --- | --- |
| miR-7-5p mimic | Sense 5′-UGGAAGACUAGUGAUUUUGUUGUU-3′ |
|  | Antisense 5′-CAACAAAAUCACUAGUCUUCCAUU-3′ |
| miR-7-5p mimic NC | Sense 5′-UUCUCCGAACGUGUCACGUTT-3′ |
|  | Antisense 5′-ACGUGACACGUUCGGAGAATT-3′ |
| miR-7-5p inhibitor | 5′-AACAACAAAAUCACUAGUCUUCCA-3′ |
| miR-7-5p inhibitor NC | 5′-CAGUACUUUUGUGUAGUACAA-3′ |

Notes: miR, microRNA; NC, negative control.
